# From diversity to disease: unravelling the role of enteric glial cells

**DOI:** 10.3389/fimmu.2024.1408744

**Published:** 2024-06-18

**Authors:** Sneha Santhosh, Lisa Zanoletti, Lincon A. Stamp, Marlene M. Hao, Gianluca Matteoli

**Affiliations:** ^1^ Department of Chronic Diseases, Metabolism (CHROMETA), Translational Research Center for Gastrointestinal Disorders (TARGID), KU Leuven, Leuven, Belgium; ^2^ Department of Anatomy and Physiology, The University of Melbourne, Parkville, VIC, Australia; ^3^ Department of Biology and Biotechnology “Lazzaro Spallanzani”, University of Pavia, Pavia, Italy; ^4^ Leuven Institute for Single-cell Omics (LISCO), KU Leuven, Leuven, Belgium

**Keywords:** enteric glia, gastrointestinal diseases, homeostasis, immune cells, enteric glia communications, enteric glia diversity, enteric nervous system

## Abstract

Enteric glial cells (EGCs) are an essential component of the enteric nervous system (ENS) and play key roles in gastrointestinal development, homeostasis, and disease. Derived from neural crest cells, EGCs undergo complex differentiation processes regulated by various signalling pathways. Being among the most dynamic cells of the digestive system, EGCs react to cues in their surrounding microenvironment and communicate with various cell types and systems within the gut. Morphological studies and recent single cell RNA sequencing studies have unveiled heterogeneity among EGC populations with implications for regional functions and roles in diseases. In gastrointestinal disorders, including inflammatory bowel disease (IBD), infections and cancer, EGCs modulate neuroplasticity, immune responses and tumorigenesis. Recent evidence suggests that EGCs respond plastically to the microenvironmental cues, adapting their phenotype and functions in disease states and taking on a crucial role. They exhibit molecular abnormalities and alter communication with other intestinal cell types, underscoring their therapeutic potential as targets. This review delves into the multifaceted roles of EGCs, particularly emphasizing their interactions with various cell types in the gut and their significant contributions to gastrointestinal disorders. Understanding the complex roles of EGCs in gastrointestinal physiology and pathology will be crucial for the development of novel therapeutic strategies for gastrointestinal disorders.

## Introduction

The enteric nervous system (ENS) is an extensive neural network distributed throughout the gastrointestinal tract, running from the oesophagus to the colon (1–3), and is essential for the control of digestion. It is comprised primarily of two cell types: enteric neurons and enteric glial cells (EGCs). These cells are organized into ganglia located in two concentric and interconnected plexuses embedded in the gut wall, which locally regulate the function of the gut smooth muscle, blood vessels, glands, and immune cells (4).

EGCs are a group of peripheral neuroglia associated with the cell bodies and neurites of enteric neurons throughout the whole gastrointestinal tract. These cells are found both within the ganglia of the ENS, where they surround enteric neurons and in the muscle layers and mucosa, where they are associated with neuronal processes. In all species examined, they appear to outnumber enteric neurons, however, the exact quantity differs depending on the region of the gut examined (5, 6).

In vertebrates, the majority of enteric neurons and glia arise from neural crest cells, which migrate into the gut during embryonic development (7–9). ENS development is orchestrated by many signalling pathways, whose timing and intricate regulation are crucial to establishing a fully functional neural and glial network. While significant progress has been made in understanding pathways involved in differentiation, the knowledge of how bipotent progenitors make the critical decision to become either neurons or glia is still limited. A recent study has shown that enteric neuron vs glia fate determination follows a branching model, where neurogenic differentiation branches off from a linear default gliogenic fate (10).

In the adult gut, there are several different subtypes of EGCs, which have been classified by their location and morphology in the gut (11, 12) and more recently, by their various gene expression patterns (13–15). However, as with enteric neurons, there is currently no consensus on how these different classification schemes relate (16). Recently, there has been growing interest in EGC heterogeneity and plasticity. Indeed, it has been demonstrated that specific intestinal environmental cues drive EGCs’ phenotype and functions, resulting in subpopulations of glia with unique characteristics within the different intestinal regions (12, 17). However, our understanding about regional or local glial heterogeneity, contributions of glial specializations to the various digestive functions, and whether functional plasticity exists between the glial subpopulations remains limited.

EGCs exhibit remarkable similarities to their counterparts in the central nervous system (CNS). Although they have different embryological origins, EGCs have been shown to share many morphological and functional characteristics with astrocytes, the main glial cell type of CNS (18–20) They also share many common markers, with both EGCs and astrocytes expressing glial fibrillary acidic protein (GFAP) and S100 calcium-binding protein B (S100β). However, robust astrocyte markers, such as glutamate receptor 1 (Glast-1) and aldehyde dehydrogenase family member L1 (Aldh1L1), are not detected in EGCs (21, 22). Transcriptomic analysis has revealed that EGCs are most similar to Schwann cells (23), satellite glia, and interestingly, oligodendrocyte progenitor cells of the CNS (13).

Since their initial discovery in the gut, various studies have highlighted the substantial role of EGCs in digestive physiology. They support myenteric and submucosal neurons and regulate the activity of intestinal neural circuits (5). More recently, EGCs have been shown to act as the neural stem cells of the gut. Although they appear to be generally quiescent (24), subset of EGCs can differentiate into enteric neurons when there is ENS damage (10, 15, 25). In addition to their conventional role of supporting enteric neurons, recent studies have unveiled their direct involvement in several biological processes, such as gut inflammation and defence (26–28). Furthermore, EGCs have emerged as a significant player in diseases, such as neuroinflammation, cancer, and gut infections, modulating inflammation and immune response (29–32).

In this review, we highlight the novel insights regarding the diversity of EGCs and their role in gastrointestinal disorders.

## Enteric glia are a heterogenous population

**Figure 1:**
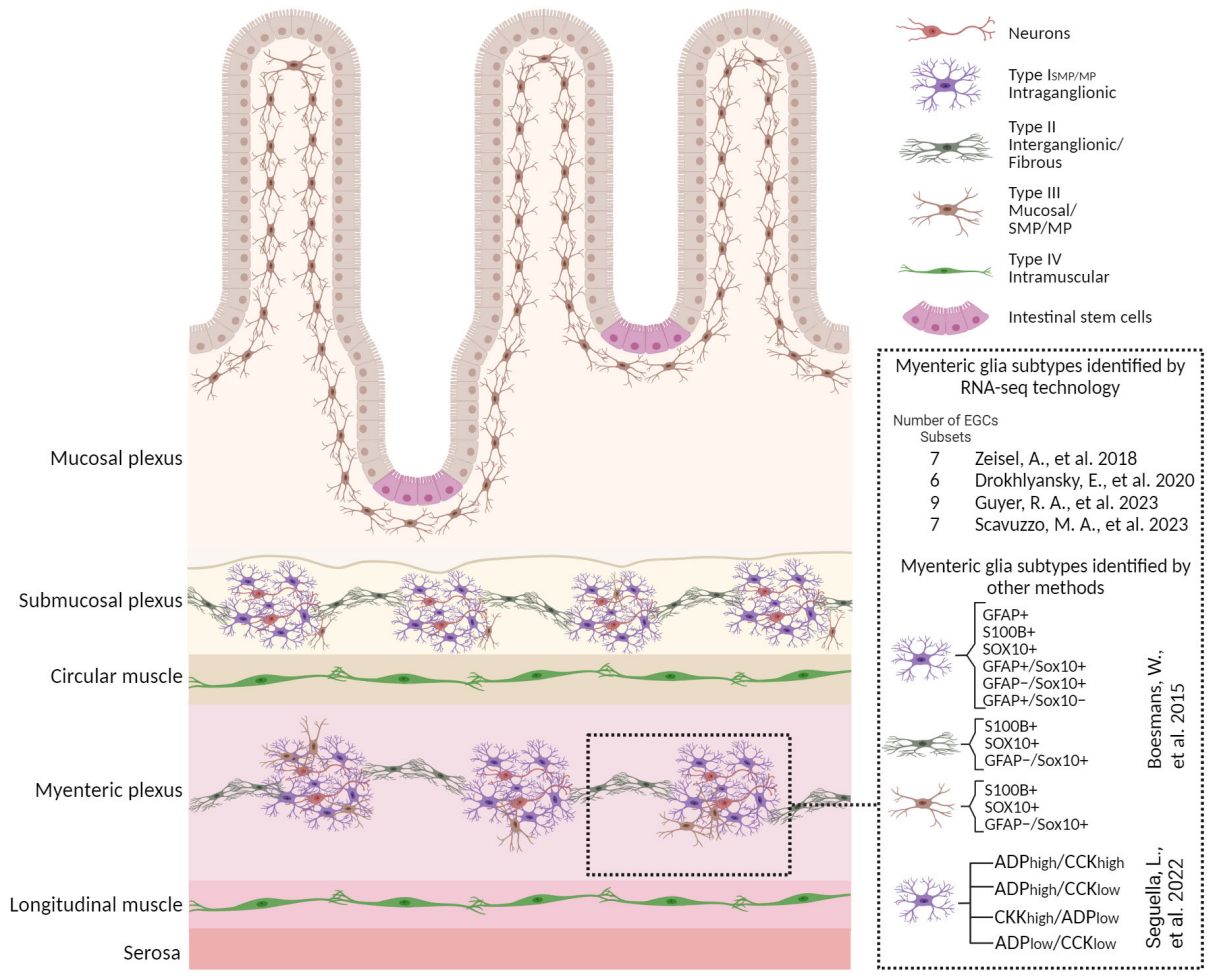
Enteric glial cell heterogeneity. Schematic overview with a magnified view of small bowel cross section illustrating the glial heterogeneity identified by various methods including scRNA-seq technologies and their distribution in different anatomical layers. Four morphological subtypes identified in rodent models are depicted here. An overview of different classifications of the myenteric glia identified by various methods are also shown along with corresponding studies. Details are described in the section “Enteric glia are a heterogeneous population” in the main text. Figure was created with BioRender.com.

Diverse populations of EGCs are distributed across distinct layers of the gut wall, reflecting their specialized adaptations to local microenvironments within the digestive tract (**Figure 1**). While this diversity implies spatial and functional specialization, elucidating the intricate interplay between niche-specific signalling cues and lineage determinants in driving EGC heterogeneity has not yet been extensively explored.

The current classification includes four EGC subtypes, depending on their morphology and anatomical location within the intestinal wall and the enteric ganglia (11, 12, 33, 34). In some cases, this increases to six subtypes when myenteric and submucous EGCs are further separated (5). Type I EGCs surround neuron cell bodies with short and irregularly branched processes within the myenteric (Type I_MP_) and submucosal plexuses (Type I_SMP_). In contrast, fibrous glia (Type II) present long processes that connect myenteric ganglia. The multipolar Type III EGCs are found in the mucosa (Type III_MUCOSA_) and the myenteric and submucosal plexuses (Type III_MP/SMP_). The Type IV EGCs, which are elongated, reside in the smooth muscle layers (5, 33). Interestingly, in a recent study, Scavuzzo and colleagues identified two additional populations with triad and bipolar shape (35). However, this classification does not capture the complete functional heterogeneity among intestinal regions or even within the major subtypes themselves.

It is now evident that EGCs have distinct responses to neuromodulators in the duodenum and colon, suggesting functional diversity across intestinal regions. This classification based on the functional response profile to adenosine diphosphate (ADP) and cholecystokinin (CCK) delineates 4 unique subpopulations, whose heterogeneity is probably associated with differing mechanisms of communication with neurons (17). Moreover, molecular and functional data suggest added complexity within and between the major glial subtypes. Commonly used markers to label EGCs include GFAP, the Ca^2+^ binding protein S100β, proteolipid protein 1 (PLP-1), and the transcription factor SRY-Box Transcription Factor 10 (SOX10) (12, 23). While no single glial marker identifies a particular enteric glial subtype, there does appear to be some regional and cell-type specificity in marker expression. For example, the expression of GFAP is dynamic and varies depending on the glial state (29) and subtype (12). About 20%–40% of glial cells that are extra-ganglionic express only GFAP (23). Additionally, the detection and distribution of GFAP+ cells in immunolabeling experiments may be limited by the antibody’s ability to recognize one or more GFAP isoforms (36–39).

To better delineate and characterize unique EGC populations in the gut, emerging techniques such as single-cell RNA sequencing (scRNA-seq) have been employed. The first published study in 2018, identified seven distinct EGCs sub-types, including one proliferative population (13) in the mouse small intestine myenteric plexus of 3-week-old mice. More recently, using a different transgenic strain to specifically isolate most of the EGCs resulted in the identification of 9 transcriptionally different clusters at postnatal day 14 (P14) (15). By contrast, in the human colon, six clusters of EGCs have been identified, including 3 subsets from patients with colorectal cancer (CRC) (14). Furthermore, Scavuzzo and colleagues demonstrated that EGCs share many genes with glia in CNS and peripheral nervous system (PNS) but in unusual combinations, conferring them a distinct identity compared to all other glia in the body. Hence, they made use of single-nucleus RNA-sequencing and identified 7 distinct molecular classes of EGCs with different signatures between duodenal and colonic EGCs (35). They identified a functionally specialized biosensor subtype of EGCs, called “hub cells”. These hub cells can impact the function of enteric neurons and thus regulate contractions in the intestine and gut physiology through the mechanosensory ion channel PIEZO2 (35). Currently, a major issue plaguing the field is the lack of intercalation between the transcriptomic and morphological phenotyping of EGCs. While we can hypothesize how the different functions of EGCs relate to their position in the gut, there is currently no information on how this relates to their gene expression characteristics.

## Enteric glia as communicators in the gut

**Figure 2:**
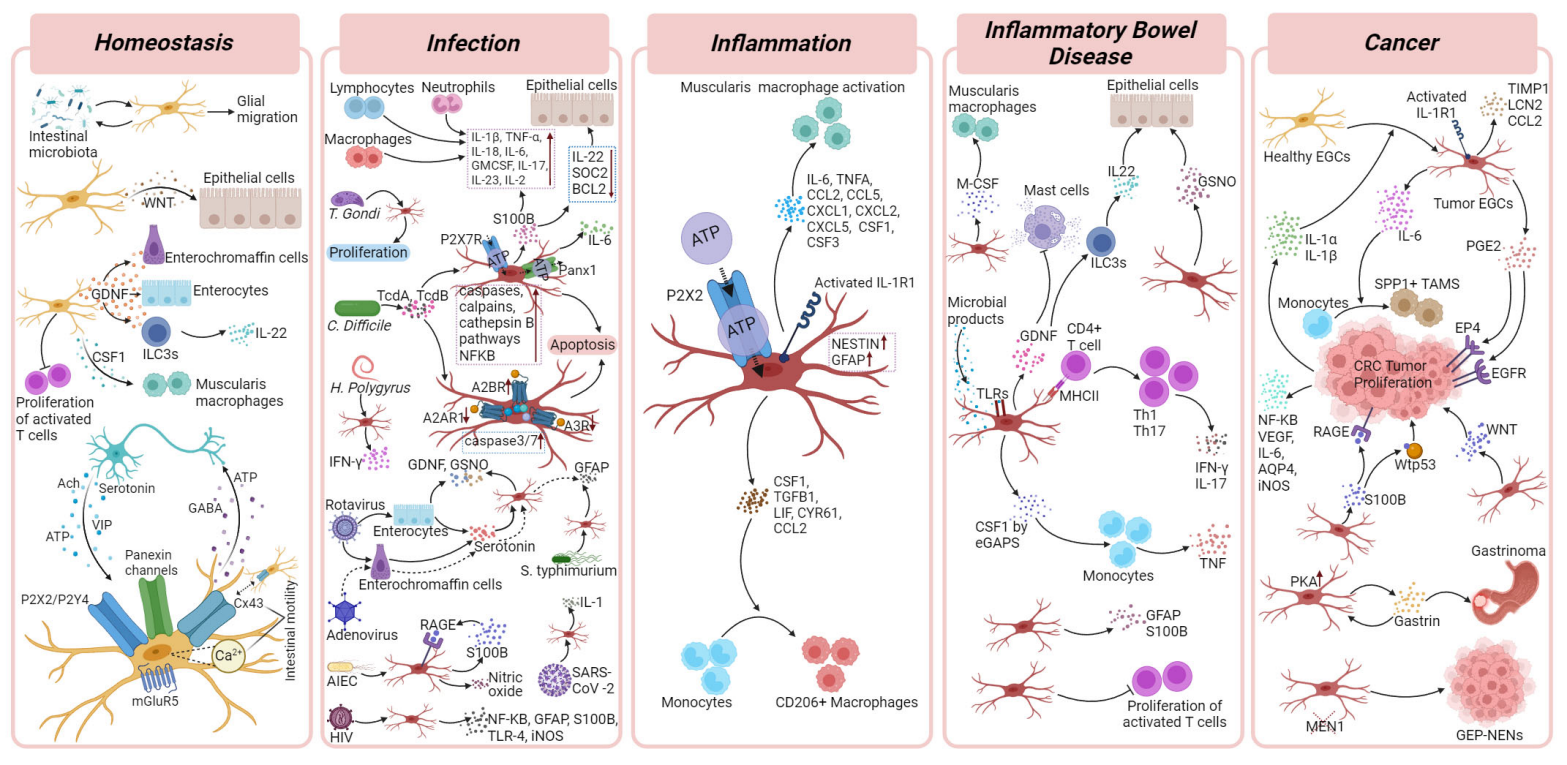
Enteric glial cells functions in homeostasis and gastrointestinal disorders. Cellular and molecular mechanisms of EGCs and their crosstalk with surrounding cells in homeostasis, infection, inflammation, inflammatory bowel diseases and cancer. Details are described in corresponding disease sections of the main text. Figure was created with BioRender.com.

The strategic positioning of EGCs within all the layers of the intestinal wall suggests their capacity for engaging in crosstalk with diverse cell types. Not only has the bidirectional communication between EGCs and enteric neurons via neurotransmitters and gliotransmitters been widely demonstrated (40–43), but recent studies also suggest how EGCs communicate with enterocytes, the stem cell niche, and enteroendocrine cells (5, 40, 41, 44). The data collected so far indicated these cells as important players in gastrointestinal homeostasis and gut motility, even though their exact contribution is still under intense investigation. There is also evidence that enteric glia communicate with immune cells to maintain homeostasis, however, this has mostly been studied in the context of disease, and therefore are described in the Infection and Inflammation sections below (**Figure 2**).

### Communication with neurons

Since 1972, it has been hypothesized that communication flows between enteric neurons and glia when presynaptic specialization contacts were observed on EGCs in the myenteric plexus of the guinea pig ileum (45). Early studies showed that EGCs expressed and utilized machinery required for classical neurotransmission, including metabotropic glutamate receptor 5 (mGluR5) (46), and purinergic receptors P2Y4 (47), and P2X7 (48), and exhibited calcium transients (49, 50). However, it was not until 2009 that two studies showed that stimulation of enteric neurons triggered Ca^2+^ responses in EGCs (51, 52), which was mediated by adenosine triphosphate (ATP). It is now well known that EGCs respond to many neurotransmitters, including acetylcholine (ACh) (42, 53), serotonin (5-hydroxytryptamine,5-HT) (53), and vasoactive intestinal peptide (VIP) (54). Apart from synaptic communication, it has also been shown that neurons communicate with EGCs adjacent to their cell bodies via pannexin channels (43, 55). There is also communication between EGCs through gap junctions formed by connexin-43 (Cx43) hemichannels (56) and release of “gliotransmitters” that are likely to influence enteric neuron function, including ATP, gamma-aminobutyric acid (GABA), and prostaglandins (56–58). The intricacies of these enteric neuron-glia signalling pathways have recently been reviewed (59).

While enteric neurons primarily control gut motility, the importance of EGC signalling has been highlighted in several key *in vivo* studies. An inducible mouse knockout model of EGCs also revealed that gut transit is delayed in female mice but not male mice, where distinct alterations in colonic motility were observed (60). Inhibition of glia-glia signalling by blocking gap junctions resulted in slowed gut motility (57). Interestingly, this slowed transit was mimicked by aging, where expression of Cx43 became dysregulated, and reduced EGC Ca^2+^ responses were observed (57). Further expansion of this study showed that alterations in Cx43 expression and Ca^2+^-mediated exocytosis in EGCs influenced colonic motility (41, 61). Although there are differences in specific results of some of these studies, which are likely attributed to the mechanisms employed (glial inhibition vs. knockout) as well as the CRE-driver lines used (e.g., PLP1 vs. GFAP), it is clear that EGCs have a distinct role in the control of gut motility. Imaging studies have also shown that Ca^2+^ activity in EGCs correlates with contraction complexes in the mouse colon (62). In addition, the use of chemogenetic systems has highlighted that stimulation of EGC activity also increased gut motility, both in *ex vivo* preparations of the gut and *in vivo* (42). More recently, a single nucleus RNA-sequencing study has identified a subtype of enteric glial cells that resemble pancreatic hub cells, which expresses the mechanosensitive receptor, Piezo2. Knockout out *Piezo2* in EGCs resulted in alterations in gut motility *in vivo (* 35).

### Communication with epithelium

The intestinal epithelium acts as a physical and biochemical barrier that separates host tissue from the contents of the gut lumen to maintain intestinal homeostasis. Given the anatomical proximity of EGCs and epithelial cells in both the small and large bowel, several studies have attempted to define the effect of the enteric glia on the intestinal epithelial barrier. Two key early studies using murine depletion models showed that EGCs play a vital role in controlling intestinal epithelial barrier function, with their ablation leading to severe intestinal inflammation (63, 64). However, more recent studies using different transgenic mouse models suggest that the role of EGCs are not as crucial (60). An early model of glial ablation by Bush et al. (63) involved driving the herpes simplex virus thymidine kinase (HSV-TK) under the regulation of the GFAP promoter (Gfap-HSV-Tk mice), and application of ganciclovir to ablate GFAP-expressing EGCs (63). However, it has been suggested that much of the inflammatory damage may have been due to the processing of ganciclovir used to ablate GFAP+ EGCs, which resulted in non-cell autonomous cytotoxic effects. Another earlier study used T-cell targeting to ablate EGCs by expressing the hemagglutinin gene under the GFAP promoter (64). These two studies promoted a significant amount of research focusing on the interactions between EGCs and the intestinal epithelium, many using different cell lines and cell culture models. More recently, ablation of EGCs using transgenic expression of diphtheria toxin (subunit A) under the PLP1 promoter did not result in the same excessive inflammatory effects. The authors described a rather limited impact on intestinal epithelial barrier functions; instead, they observed increase in gut motility in female mice (60). Interestingly, a similar study using the expression of diphtheria toxin under the GFAP promoter resulted in an acute reduction of the epithelial stem cell niche, suggesting a specific nurturing role for GFAP-expressing EGCs (65). These studies highlight the importance of interpreting the use of various mouse models in understanding EGC function, with the different combinations of promoters and cell ablation mechanisms all leading to different downstream effects. While EGCs may not be as essential in the maintenance of the intestinal epithelial barrier, communication between EGCs and intestinal epithelial stem cell niche has been recently described (65). Apart from mesenchymal and paneth cells, EGCs also secrete WNT ligands to regulate intestinal stem cell regeneration during homeostasis and inflammation. From a mechanistic standpoint, GFAP-expressing glial cells are supplying WNT ligands, which are vital for sustaining the intestinal stem cell niche. Under homeostatic conditions, depletion of GFAP-expressing EGCs results in a transient decline in stem cell numbers, which are promptly restored to their usual levels shortly thereafter. This rapid recovery is likely due to the activation of an alternative WNT source (65, 66).

Mucosal EGCs may also maintain gastrointestinal homeostasis by communicating with enterocytes and enteroendocrine cells. The presence of glial cell-derived neurotrophic factor (GDNF) contributes to the development and maturation of enterocytes, thereby ensuring the integrity of the gastrointestinal epithelial barrier (67). However, as many other cells in the gut produce and release GDNF, this impact is likely not restricted to EGCs. The crosstalk between mucosal glia and enteroendocrine cells also suggests a potential for glial influence on gastrointestinal hormone release (68). In addition, EGCs release factors like epidermal growth factor (EGF) and transforming growth factor β (TGF-β) isoforms in response to inflammation or injury (69, 70). The exact role of EGCs in these intricate communications will be an interesting field of future study.

### Communication with intestinal microbiota

While intra-ganglionic glia is surrounded by a connective tissue barrier (71), mucosal glia located in the lamina propria have greater exposure to luminal microbes. There is also evidence of interaction between the intestinal microbiota and mucosal glia. Signals from luminal microbes may influence glial migration within the gut, especially from the myenteric plexus to the lamina propria postnatally, stabilizing after weaning in mice due to microbial and immune changes. Thus, EGCs do not appear in the lamina propria of germ-free mice (72). Additionally, recent studies showed that antibiotic treatment reduces the number of enteric glial cells in the mucosa and in the ileal myenteric plexus (72, 73). However, xenografts of human foetal intestines maintained for months in immuno-compromised mice exhibit a mucosal glial network even after antibiotic treatment, suggesting differences in glial responses between humans and mice, potentially due to distinct glial gene expressions and the microbiota diversity’s impact on their development (74).

## Enteric glia in gastrointestinal disorders

The gastrointestinal tract relies on the complex interplay between the nervous and immune systems to sense, detect, decode, and react to various environmental stimuli. EGCs are now being recognized as one of the regulators of intestinal stem cell proliferation, immune function, defence mechanisms, and tissue repair. Given their role in maintaining intestinal homeostasis alongside other cells, EGCs are also thought to play a crucial role in neuropathology (34, 75, 76). This is particularly significant in disorders of gut-brain interaction (DGBIs; formerly known as functional gastrointestinal and motility disorders, FGIDs), which are characterized by dysmotility, pain, and age-associated decline in gastrointestinal neuromuscular function (77–80). EGCs influence pathological mechanisms in intestinal diseases by modulating neuroplasticity and immune responses (29, 81, 82). Due to their ability to respond to microenvironmental cues through the expression of immunomodulatory molecules, EGCs have gained recognition for their role in modulating the mucosal immune response (83). Recent research breakthroughs have illuminated the intricate cellular dialogues and molecular mechanisms by which EGCs shape intestinal health and disease outcomes.

### Infections

Due to constant exposure to the external environment and the potential for acquiring infections through food and water, the intestinal tissue is equipped with a vast immune system, fully prepared to possibly fight pathogens. This complex defence mechanism relies not only on immune cells but also on the active participation of various tissue cells, including those of the nervous system. Neurons and glial cells within the ENS interact with immune cells, influencing their activity and ensuring a fast and coordinated response to infection and inflammation.

EGCs are fully equipped with pattern recognition receptors that enable them to respond to bacterial and inflammatory stimuli using surface proteins, cytokine/chemokine receptors, and the secreted proinflammatory factors, highlighting their crucial role in infections. They detect micro-organisms, damage-associated molecular patterns (DAMPs), pathogen-associated molecular patterns (PAMPS), and signals from immune cells via functional pattern recognition receptors, such as toll-like receptors (TLRs), nod-like receptors (NLRs), cytokine receptors (e.g., Interferon-gamma receptor(IFNγR)), as well as related factors needed to respond to inflammatory triggers (e.g., NF-κB, MYD88, STAT1, STAT3, etc.) (72, 83). Upon stimulation, EGCs release cytokines and chemokines that contribute to either pro- or anti-inflammatory conditions (33, 41, 83–85). Initially, *in vitro* studies have unravelled the mechanisms underlying the EGCs ‘response to inflammatory stimuli (84, 85). However, it is now evident that EGCs can also respond to challenges within the tissue *in vivo (* 29, 82, 86, 87).

During infection, EGCs acquire a distinct molecular state similar to astrogliosis (88) involving cell cycle entry and GFAP upregulation, enabling them to exert local immunomodulatory effects on surrounding cells (87, 89–91). This adaptive capacity allows EGCs to play a significant role in gastrointestinal infections.

Recently, EGCs have gained attention for their potential involvement in *Clostridium difficile* (*C. difficile*) infection, the most common cause of antibiotic-associated diarrhoea and colitis. This bacterium, typically a minor component of the gut microbiota in 1–3% of adults, can proliferate in the large intestine following the disruption of commensal bacteria by broad-spectrum antibiotics. *C. difficile* toxin A (TcdA) and toxin B (TcdB) affect various cell types in the intestine, including enterocytes, colonocytes, and enteric neurons. These toxins induce glucosylation of Rho GTPases, leading to cytopathic and cytotoxic effects, as well as inflammation. Studies have shown that EGCs are particularly susceptible to TcdB. Early effects like cell rounding and Rac1 glucosylation, resulting in cell cycle arrest are observed in rat-transformed EGCs treated with this toxin (92). Furthermore, TcdB has been found to induce apoptosis in EGCs *in vitro* by activating signalling pathways mediated by caspases, calpains, and cathepsin B. This activation of apoptosis was enhanced in the presence of the pro-inflammatory cytokines Tumour Necrosis Factor-alpha (TNF-α) and IFN-γ. Notably, the ability to activate three different apoptotic signalling pathways could be an advantageous strategy adopted by *C. difficile* to overcome cell resistance mechanisms (93, 94). Moreover, a recent study reported that Pannexin-1 (Panx1) channel, known for its role in intestinal inflammation and IBD, is increased in the intestinal tissue of mice during *C. difficile* infection. Panx1 which activates P2X7 receptors, contributes to the effects of *C. difficile* toxins in EGCs, promoting caspase-3/7-mediated cell death and Interleukin-6 (IL-6) over-expression (95). Adenosine receptors also act as key regulators of EGC response to TcdA and TcdB, pointing out A2B activation as an important mechanism in inducing glial apoptosis (96).

On the other hand, *in vivo* studies detected an increase of S100β in both colon tissues and faecal samples from patients with *C. difficile* infection, as well as in colon tissues of mice infected with *C. difficile*. S100β modulates the inflammatory response during CDI, by upregulating proinflammatory mediators such as IL-1β, IL-18, IL-6, Granulocyte macrophage colony-stimulating factor (GM-CSF), TNF-α, IL-17, IL-23, and IL-2, that promotes immune cells recruitment and downregulating the protective mediators, including SOCS2, IL-22, and BCL-2 that lead to epithelial damage (97). In EGCs, S100β upregulation induces IL-6 expression via activation of the RAGE/PI3K/NFκB signalling pathway. Inhibition of S100β activity has been shown to mitigate intestinal damage and diarrhoea caused by *C. difficile* toxins, suggesting that targeting the production of S100β in EGCs could be a potential therapeutic strategy for managing the effects of CDI on the gastrointestinal system (97).

EGCs are also involved in *Toxoplasma gondii* (*T. gondii*) infection. *T. gondii* affects up to one-third of the global population and is contracted by consuming raw meat containing tissue cysts or food contaminated with oocysts. Once in the gastrointestinal tract, *T. gondii* rapidly proliferates and crosses the intestinal barrier, triggering a local immune response. Research indicates that infection with various strains of *T*. *gondii* induces alterations in neuronal subpopulations and epithelial cells across various segments of the gastrointestinal tract (98). In a study using Wistar rats to evaluate the kinetics of neuronal and glial responses after ingestion of sporulated oocysts, significant effects on the populations of enteric neurons and EGCs were observed starting 72 hours post-infection. While the total neuron population was decreased, the number of EGCs in the myenteric plexus remained unaffected. In contrast, both the EGCs and total neuron populations in the submucosal plexus decreased, although the reduction in EGCs was less pronounced compared to neurons. Overall, an increase in the EGC/neuron ratio was noted. This suggests a protective role for EGCs against infection (99)..

Helminth infections in mice induce an active phenotype in EGCs along with an upregulation of an IFN-γ gene signature. ScRNA-seq was performed on the smooth muscle layers of mice infected with third stage *Heligmosomoides polygyrus* larvae. These larvae settle in the smooth muscle of the duodenum, causing tissue inflammation locally and multicellular granulomatous infiltrates. The single-cell transcriptomics analysis emphasized that specifically inhibiting IFNγ signalling in glial cells leads to the widespread activation of pro-inflammatory transcriptional programs throughout the tissue, identifying this pathway as fundamental in restoring tissue integrity after helminth infection. Moreover, CXCL10 has been pointed out as a critical mediator of tissue repair downstream of the IFNγ-induced activation of EGCs. This demonstrates that IFNγ–EGC–CXCL10 axis plays a fundamental role in immune response and tissue repair after infectious challenge (87).

Rotaviruses, transmitted via the faecal-oral route, rank among the primary reasons for diarrheal illness in infants and young children (100). In surprising contrast to the common enteric bacterial infections, during diarrhoea, rotavirus infections do not affect intestinal permeability in humans (101–104)and mice (103, 105). Studies link rotavirus infection with the activation of EGCs, which are triggered by infected enterocytes that produce serotonin. S-nitrosoglutathione (GSNO) and GDNF from both EGCs and enterocytes protect the gut barrier from infection, while observations also suggest possible communication between infected enterocytes, enterochromaffin cells, enteric neurons and EGCs as they are all seen in close proximity (103). In line with these findings, it is also reported that rotavirus-infected enterochromaffin cells in mice secrete serotonin, which subsequently upregulates GFAP expression in the EGCs (103, 106). A similar mechanism was also observed in the case of human adenovirus-41 (HAdV-41) infection, where EGCs had high expression of GFAP under the influence of serotonin released from enterochromaffin cells that expressed HAdV-41-specific coxsackievirus and adenovirus receptor (CAR) (106–108). EGC activation is also induced during the Human Immunodeficiency Virus (HIV) infection. Studies in rats found that viral HIV-1 Trans activating factor (HIV-1 Tat) protein induces diarrhoea modulated by activation of EGCs. This activation involved high expression of NF-κB, GFAP, S100B, TLR-4 and inducible nitric oxide synthase (iNOS) which causes a neuroinflammatory response. This local response can activate EGCs in the spinal cord and brain cortex through the expression of Cx43, causing inflammation associated with significant cognitive decline (106, 109).

Additionally, *Salmonella typhimurium* (*S. typhimurium*), an intracellular pathogen, affects the ENS and results in the death of enteric neurons (110, 111). Although not extensively studied, research suggests a potential role of EGCs in barrier function during Salmonella infection. A study exploring the involvement of EGCs in barrier function in inflammatory bowel syndrome (IBS) used biopsies from the colon of IBS patients and healthy controls to show that expression of GFAP significantly correlated with the passage of Salmonella, but not with Escherichia coli (E. coli) (112). A potential reason could be that many strains of *E.coli* are commonly found in the intestines of humans and other animals. Most *E. coli* strains are harmless or beneficial. Indeed, recent evidence also indicates that human EGCs can distinguish between pathogenic and probiotic bacteria via TLR activation (21). However, there are also pathogenic strains of *E.coli*, including *O157:H7*, which produces toxins that can lead to bloody diarrhoea and abdominal cramps (113). The *Adherent-invasive E. coli* (*AIEC*) strain is a pathobiont associated with Crohn’s Disease, which prefers to grow in inflammatory conditions. Even though the role of EGCs in *E. coli O157:H7* is unknown, pathogenic *AIEC* are known to activate human EGCs and induce cFos and MHC II expression. TLRs on human EGCs detect AIECs, leading to S100B overexpression and a reactive phenotype. S100B interacts with RAGE on EGC surfaces, and the S100B/RAGE complex may interact, directly or indirectly, with MyD88, a downstream regulator of the TLR signalling pathway, leading to iNOS expression and nitric oxide release through NF-κB-mediated pathways, sustaining nitric oxide signaling (21).

John Cunningham Virus is a polyomavirus commonly found in humans (114). While it often remains dormant and asymptomatic in healthy individuals, John Cunningham Virus can cause serious complications in individuals with weakened immune systems (114, 115). John Cunningham Virus affects the EGCs in the myenteric plexus in patients with chronic idiopathic intestinal pseudo-obstruction (CIIP). A Study involving 10 CIIP patients and 61 controls (colon and ileum from patients with uncomplicated colon cancer) revealed neurogenic impairment and neuropathy in CIIP patients. John Cunningham Virus T antigen DNA was detected in the myenteric plexuses of most CIIP patients but not controls and the John Cunningham Virus viral protein1 co-localized with glial fibrillary acidic protein, a marker of EGCs suggesting that the virus infect EGCs and use them for replication (32, 106).

Additionally, there have been hypotheses proposing that SARS-CoV-2-related diarrhoea and gastrointestinal dysfunction in COVID-19 patients may involve activation of the ENS and EGCs, as activated EGCs are known to release IL-1 and other inflammatory mediators typically observed in COVID-19 patients (106, 116–119).

Altogether, EGCs play diverse and dynamic roles in the host-defence against gastrointestinal infections, encompassing immune modulation, neuroimmune interactions, antimicrobial defence, barrier function, and tissue repair. Further research to elucidate the mechanisms underlying EGC-mediated responses to infection may offer insights for prevention and treatment of infectious diseases.

### Intestinal inflammation

Intestinal inflammation triggers a complex immune response by recruiting innate and adaptive immune cells, creating a highly coordinated defence within the gastrointestinal tract. Accumulating evidences have now confirmed that EGCs play a crucial immunoregulatory role in these inflammatory conditions, capable of interacting with both the innate (87, 90, 120)) and adaptive immune systems (91, 121). EGCs are able to quickly respond and adapt to inflammatory cues, and acquiring a reactive state named “gliosis” characterized by alterations in molecular composition, structure, and function. This transformation into reactive EGCs is influenced by various factors, including the injury’s nature and severity, as well as the specific glial subtype (29). Enteric gliosis is also part of an intestinal immune response that occurs upon abdominal surgery that leads to impaired motility in postoperative ileus. An extensive investigation that involved *in vivo*, *ex vivo*, and *in vitro* analysis in mice and human specimens, revealed that the activation of EGCs in this process is induced by ATP released during tissue damage triggering purinergic P2X2 signaling (122).

Previous studies also showed that the activation of IL-1 receptor type 1 (IL1R1) specifically on enteric glia protects mice from development of postoperative ileus after intestinal manipulation (123). Activation of IL-1R1 in EGCs typically results in the release of inflammatory mediators in postoperative ileus such as IL-6 and CCL2 that activates immune cells (120, 122–124). Similar findings have also been validated in patients, undergoing abdominal, further supporting the crucial role of EGCs in intestinal inflammation (122, 125). However, activation of EGCs during muscularis inflammation also results in protective mechanisms favouring recovery of dysmotility (125). Interestingly, EGC activation after muscularis externa damage results in production of CCL2 and CSF1 which in turn stimulated monocyte infiltration and differentiation in anti-inflammatory CD206+ macrophages, respectively (120). Furthermore, CSF1-CSF1R signalling has been demonstrated to be crucial for the differentiation of monocytes into neurotrophic macrophages favouring proliferation of EGC during muscularis inflammation (120). Taken together, these studies indicate the inevitable role of EGCs in inflammation and their potential to be targeted for novel therapeutic strategies for inflammatory disorders.

### Inflammatory bowel disease

IBD refers to chronic inflammatory disorders affecting the gastrointestinal tract, whose prevalence is rapidly increasing in Western countries, affecting more than 2 million Europeans and 1,5 million North Americans (126). Crohn’s disease (CD) and ulcerative colitis (UC) are the main disorders belonging to IBD, affecting different segments of the GI tract but sharing common features, mainly chronic inflammation and abnormal intestinal immune response. Common symptoms include abdominal pain, diarrhoea, lower gastrointestinal tract bleeding, weight loss, and fatigue, leading to reduced quality of life (127). Although the aetiology of IBD is still not completely understood, a central player in the development and progression of IBD is the immune system, which may mistakenly trigger an exaggerated inflammatory response against harmless substances (i.e. food and bacteria) in genetically predisposed individuals. This abnormal immune response involves various immune cells, including T cells, B cells, and macrophages. The chronic inflammation damages the intestinal tissues and disrupts their normal functions, leading to the characteristic symptoms of IBD (127). Notably, 70% of IBD patients do not respond properly to the first-line therapy, implying that the immune cells are not the only players in the pathogenesis of this disease. Moreover, the course of IBD is unpredictable, with periods of active inflammation known as flare-ups and remission when symptoms subside.

Psychological stress exerts a profound impact on IBD, with numerous studies suggesting that stressful life events can exacerbate IBD flares (128–130). In line, recent research identified the ENS as a relay between psychological stress and inflammation in the gut, highlighting the importance of the gut-brain axis (131). The signalling pathway of glucocorticoids from the adrenal gland influences the impact of stress on IBD. During periods of stress, an inflammatory subset of EGCs is generated, termed enteric glia associated with psychological stress (eGAPS). These eGAPS then produce CSF1, which triggers the production of TNF by monocytes. This TNF production exacerbates inflammation, thereby exacerbating IBD (131). Altogether, ENS system plays a critical role in IBD pathogenesis.

Recent research has explored the role of EGCs in IBD, revealing that EGCs in IBD patients exhibit molecular abnormalities. Interestingly there is an increased expression of GFAP in inflamed colonic biopsies from CD and UC patients (132). In line, another glial marker, S100β, also shows roughly the same expression profile, with increasing S100β in inflamed colonic biopsies compared to healthy controls (133–136). Additionally, certain factors involved in glial cell signalling, are upregulated in patients with IBD. For instance, GDNF is upregulated in biopsies of CD and UC patients, and TLR4 is found to be upregulated in glia cultures from patients with UC (132, 134).

Considering that these molecular abnormalities may lead to functional alteration, it is likely that even the crosstalk between EGCs and other intestinal cell types may be defective in the context of IBD. DSS-induced colitis mouse models are being extensively used to study IBD. During DSS-induced colitis, EGCs and neurons are exposed to inflammatory stimuli, once intraganglionic macrophages induce degradation of the membrane surrounding the myenteric plexus (71). Studies investigating the interplay between EGCs and macrophages have also identified key mechanisms driving visceral hypersensitivity in chronic colitis (90). Pro-inflammatory signals such as IL-1β, induce glial reactivity, triggering Cx43-dependent M-CSF production via protein kinase C (PKC) and TNF-α converting enzyme (TACE) that cleaves membrane-bound M-CSF. Particularly, in a mouse model lacking glial Cx43 (*Sox10CreERT2;Cx43^f/f^*), EGCs modulate muscularis macrophages through Cx43-dependent pathways, facilitated by M-CSF production (44, 83, 90). While muscularis macrophages near the myenteric plexus are activated by microbiota to produce BMP-2, influencing enteric neuron activity (137), intestinal bacterial infection triggers activation of sympathetic ganglia, shifting nearby macrophages to a protective functional profile (89).

In chronic inflammation, EGCs also interact with innate lymphoid cells (ILCs), which make part of the innate immune system and are regulated by numerous inflammatory mediators, such as neuropeptides, hormones, eicosanoids, and cytokines (138–141). The complex interplay of glia, ILC3, and epithelial cells is known to modulate intestinal homeostasis, inflammation, and plays a role in defence against certain bacterial infections (89). The primary ILC3 pathway involves integrating dendritic cell-derived IL-23 to produce IL-22, dependent on the STAT3 transcription factor (89). EGCs integrate microbiota and tissue cues through MYD88 and regulate a group of ILC3s, which express the neuroregulatory receptor tyrosine kinase RET and provide protection against DSS-induced colitis. During chronic inflammation GDNF derived from GFAP^+^ cells activate RET, leading to IL-22 induction in ILC3 and participates in controlling mucosal homeostasis (44, 89).

Simultaneously, EGCs have the capability to produce GSNO, which serves to enhance the formation of tight junctions in epithelial cells (142). There may be differences between the capabilities of different populations of EGCs. GFAP+ EGCs are crucial in regulating the permeability of the intestinal epithelial barrier, proliferation of epithelial cells, and immune cell infiltration into the ganglia (83, 142–144). However, targeting EGCs with diphtheria toxin via the PLP1 promoter did not influence epithelial cell renewal, barrier permeability, or susceptibility to DSS-induced colitis (60). EGCs have also been suggested to interact with mast cells. *In vitro* experiments have shown that GDNF inhibits enteric mast cell activation and improves the disease outcome of DSS-induced experimental colitis via JNK pathway downregulation (145). The GDNF-mediated inhibition of mast cells resulted in lower levels of TNF-α, IL-6 and tryptase expression, which would have rather increased the inflammation. The recent evidence also suggests that EGCs have significant part in the maintenance of the intestinal epithelial stem cell niche in homeostasis and chronic IBD. A recent study focusing on PLP1+ EGCs and GFAP+ EGCs found that this heterogeneity in cell populations is important in the maintenance of epithelial integrity. It is interesting that GFAP+ EGCs but not PLP+ EGCs regulate the stem cell markers and regeneration upon injury (65). This pro-regenerative effect by GFAP+ EGCs is crucial for the repair of the intestinal epithelium upon colitis. However, PLP+ EGCs might be taking over the function of maintaining intestinal functions in the absence of GFAP+ EGCs, thus compensating each other with their redundant roles (65). Delving into the intricacies of human mucosal EGC dynamics in both normal conditions and IBD, specifically UC revealed four distinct glial cell subpopulations: EGC#0–EGC#3. Interestingly, EGC#1 and EGC#2 were found to be enriched in healthy samples, while EGC#0 and EGC#3 were mainly found in individuals with UC. Comparing human gene expression signatures alongside mouse glial signatures revealed consistency between human and mouse EGCs. The human EGC#1 was similar to the mouse homeostatic Plp1High/GfapLow population and human EGC#0 resembled the GfapHigh/Plp1Low population associated with injury and inflammation (65). Studies describing glial-T cell interaction in IBD showed that EGCs isolated from CD patients inhibit the proliferation of activated T lymphocytes (121). However, the expression of glial MHC-II induced by proinflammatory stimuli have shown to enhance the activation of both B-lymphocytes and T-lymphocytes, particularly impacting T-helper cell (Th)17 and regulatory T cell subtypes to express IFN-γ and IL-17, thus maintaining immune homeostasis during inflammatory conditions (91). A similar immunosuppressive function to promote homeostasis is also observed in a study describing glia-T cell interactions in IBD; showing that EGCs isolated from CD patients inhibited the proliferation of activated T lymphocytes (121). Altogether, these data identify EGCs as a new potential target for treating IBD.

Research focusing on the effects of the isoquinoline alkaloid berberine, which has anti-inflammatory and therapeutic effects in experimental colitis, regulating neuroimmune interactions in UC revealed that when berberine was administered, disease severity was decreased and the mucosal barrier homeostasis in UC was restored. Moreover, in *in vitro* experiments using monoculture and simulated inflammatory conditions berberine exhibited direct protective effects on EGCs and other cell types such as bone marrow-derived dendritic cells (BMDCs), T cells, and intestinal epithelial cells. Furthermore, berberine could also regulate the cell-cell interactions of EGCs, intestinal epithelial cells, and immune cells in co-culture systems (146). The research conducted to date gives us an understanding of the complexity of the role of EGCs in IBD. However, further studies are to be performed for a deeper understanding of EGCs’ role in the context of IBD.

### Cancer

Recent research has unveiled compelling insights into the role of the ENS and EGCs in the pathogenesis of CRC (147). A well-known prognostic marker in colon cancer is tumoral infiltration of neural structures, i.e. perineural invasion (148, 149), which is associated with a reduced 5-year disease-free survival rate (121), presence of metastasis at the time of resection (150), and increased risk of recurrence (151, 152). As tumour cells invade the bowel wall, neurochemical alterations (153) and an increase in neural element density occur, positively correlating with tumour grading (154). Considering these premises, it is likely that tumoral and neural cells make close interactions and play a key role in carcinogenesis. In line, it has been shown that the enteric neuronal network guides tumour cell migration, with tumour epithelial cells establishing direct interactions with enteric neurons via N-cadherin and L1CAM (155). Additionally, a recent study used mice models and human tissues to demonstrate that neuronal loss of the N-Myc Downstream-Regulated Gene4(NDRG4), an important biomarker for CRC expressed by enteric neurons, is correlated with enlarged adenoma development and the release of pro-carcinogenic extracellular matrix molecules, nidogen 1 and fibulin 2, in the tumoral microenvironment (156). However, further studies in human cohorts are needed to translate these findings to a clinical level.

To date, limited attention has been dedicated to exploring whether and how EGCs may affect the development and progression of CRC. Although previous research yielded conflicting results regarding the increase or decrease in EGCs in CRC (69, 157–159), recent findings in the field confirm that EGCs are a key component of the tumour microenvironment. Particularly, three-dimensional imaging on full-thickness human colon adenocarcinomas using the iDISCO (immunolabeling-enabled imaging of solvent-cleared organs) staining, confirmed that S100β+ GFAP+ enteric glial cell bodies and projections massively infiltrate the whole tumour (31).

Notably, the tumour burden decreases following EGC depletion in azoxymethane/dextran sodium sulphate (AOM/DSS)-induced CRC mouse model and in Apc^Min/+^ mouse model of familial adenomatous polyposis, suggesting that EGCs promote tumour development at an early premalignant stage. Even though the properties of established malignant tumours were not affected by glial depletion, there is a slowing in the development of precancerous dysplastic lesions (158). Similarly, a reduction in tumour burden was also observed following the depletion of GFAP+ EGCs in an AOM/DSS-induced CRC mouse model. In these cases, glia-derived WNT ligands could also be playing a role in the proliferation of intestinal epithelium (66, 83, 158). EGCs can react to tumour epithelial cells-derived ligands IL-1 α/β and acquire an activated phenotype. Hence, EGCs elicit cancer stem cells expansion and promote tumorigenesis via a PGE2/EP4/EGFR-dependent pathway (31). However, during carcinogenesis IL-1 α/β can be produced by several other cell types, including infiltrating myeloid cells, colon epithelial cells, and stromal cells. For instance, tumour-infiltrating monocytes-derived IL-1 influences the phenotype and function of tumoral EGCs in the CRC tumour microenvironment. Subsequently, IL-1-activated EGCs produce IL-6, and promote the tumour-infiltrating monocytes differentiation into pro-tumorigenic SPP1+ TAMs. Additionally, the tumour EGCs can also present a unique reactive phenotype, with increased expression of genes correlated with astrogliosis (e.g. Lcn2 and Timp1) and immunomodulatory functions (e.g. Ccl2 and Il6). Notably, the CRC EGC phenotype is associated with poor disease outcomes, which was evident in both pre-clinical CRC mouse models and in patients with CRC (160). The expression of glial S100β is high in CRC compared to healthy tissue and this increased expression induces the downstream activation of the proliferative RAGE/MAPK/NF‐kappaB signalling pathway, resulting in a decreased expression of the pro‐apoptotic protein wtp53. Moreover, the higher expression of S100β was also associated with the up-regulation of proinflammatory and proangiogenic factors, including VEGF, IL-6, and AQP4 (159).

The glial contribution to carcinogenic progression has also been suggested in other intestinal tumours, such as duodenal gastrinomas. For example, the development of MEN1-associated gastrinoma in the submucosa might arise from EGCs through hormone-dependent PKA signaling (30). Similarly, in the context of gastroenteropancreatic neoplasms (GEP-NENs), the deletion of Men1 in GFAP+ cells led to glial cell reprogramming toward a neuroendocrine phenotype, resulting in gastric neuroendocrine hyperplasia and neuroendocrine tumours in the pituitary and pancreas (161). Overall, the recent findings on the involvement of EGCs in cancer highlight their diverse and complex roles in tumour development and progression within the gastrointestinal tract. Further elucidating the underlying molecular mechanisms may provide novel insights into the pathophysiology of gastrointestinal cancers and identify potential therapeutic targets for intervention.

## Conclusions and future directions

The ENS is commonly referred to as the “second brain” as it controls many gastrointestinal functions autonomous from the brain or spinal cord. EGCs are a key component of the ENS, regulating enteric neuronal function, as well as directly communicating with many other cell types within the gut. Recent studies have explored the multifaceted roles of EGCs in gastrointestinal physiology and pathology. However, despite significant progress, several challenges and open questions remain in understanding how EGCs function and their contributions to disease pathogenesis. One of the key challenges is unravelling the molecular mechanisms underlying EGC heterogeneity and plasticity. Even though recent studies have identified distinct EGC subpopulations, further investigations are needed to uncover the functional significance of EGC heterogeneity, particularly in the context of regional specialization and disease susceptibility. Additionally, the factors driving EGCs specialization and their implications for gastrointestinal functions warrant exploration. Understanding the precise contributions of EGCs to gastrointestinal diseases, particularly in complex disorders like cancer and IBD will open the opportunities to identify potential therapeutic targets. While studies have demonstrated the molecular mechanisms of crosstalk between EGCs and immune cells in diseases such IBD and CRC, an in-depth understanding of the mechanisms driving EGC-mediated immune modulation and resulting tissue damage or repair is necessary. Furthermore, the development of targeted therapeutic approaches leveraging EGC modulation requires a deeper understanding of their roles in disease progression and resolution. EGCs have become an immense cell of interest in understanding gastrointestinal homeostasis, particularly with recent interest in glial-immune communications. The dynamic interplay between EGCs and other cell types within the gastrointestinal microenvironment presents a complex regulatory network that demands further investigation. Building knowledge to understand the mechanisms driving these processes will provide promising avenues for innovative therapies in these diseases and gut disorders.

## Author contributions

SS: Conceptualization, Writing – original draft, Writing – review & editing. LZ: Conceptualization, Writing – review & editing, Writing – original draft. LAS: Conceptualization, Writing – review & editing. MMH: Conceptualization, Writing – review & editing. GM: Conceptualization, Writing – review & editing.

## References

[1] Furness JB. The enteric nervous system and neurogastroenterology. Nat Rev Gastroenterol Hepatol. (2012) 9:286–94. doi: 10.1038/nrgastro.2012.32 22392290

[2] Kang YN Fung C Vanden Berghe P. Gut innervation and enteric nervous system development: a spatial, temporal and molecular tour de force. Development. (2021) 148:dev.182543. doi: 10.1242/dev.182543 33558316

[3] Spencer NJ Hu H. Enteric nervous system: sensory transduction, neural circuits and gastrointestinal motility. Nat Rev Gastroenterol Hepatol. (2020) 17:338–51. doi: 10.1038/s41575-020-0271-2 PMC7474470 32152479

[4] Sharkey KA Mawe GM. The enteric nervous system. Physiol Rev. (2023) 103:1487–564. doi: 10.1152/physrev.00018.2022 PMC9970663 36521049

[5] Seguella L Gulbransen BD. Enteric glial biology, intercellular signalling and roles in gastrointestinal disease. Nat Rev Gastroenterol Hepatol. (2021) 18:571–87. doi: 10.1038/s41575-021-00423-7 PMC8324524 33731961

[6] Michel K Kuch B Dengler S Demir IE Zeller F Schemann M. How big is the little brain in the gut? Neuronal numbers in the enteric nervous system of mice, Guinea pig, and human. Neurogastroenterol Motil. (2022) 34:e14440. doi: 10.1111/nmo.14440 35929768

[7] Avetisyan M Schill EM Heuckeroth RO. Building a second brain in the bowel. J Clin Invest. (2015) 125:899–907. doi: 10.1172/JCI76307 25664848 PMC4362233

[8] Uesaka T Young HM Pachnis V Enomoto H. Development of the intrinsic and extrinsic innervation of the gut. Dev Biol. (2016) 417:158–67. doi: 10.1016/j.ydbio.2016.04.016 27112528

[9] Boesmans W Nash A Tasnady KR Yang W Stamp LA Hao MM. Development, diversity, and neurogenic capacity of enteric glia. Front Cell Dev Biol. (2021) 9:775102. doi: 10.3389/fcell.2021.775102 35111752 PMC8801887

[10] Laddach A Chng SH Lasrado R Progatzky F Shapiro M Erickson A. A branching model of lineage differentiation underpinning the neurogenic potential of enteric glia. Nat Commun. (2023) 14:5904. doi: 10.1038/s41467-023-41492-3 37737269 PMC10516949

[11] Hanani M Reichenbach A. Morphology of horseradish peroxidase (HRP)-injected glial cells in the myenteric plexus of the Guinea-pig. Cell Tissue Res. (1994) 278:153–60. doi: 10.1007/BF00305787 7954696

[12] Boesmans W Lasrado R Vanden Berghe P Pachnis V. Heterogeneity and phenotypic plasticity of glial cells in the mammalian enteric nervous system. Glia. (2015) 63:229–41. doi: 10.1002/glia.22746 25161129

[13] Zeisel A Hochgerner H Lonnerberg P Johnsson A Memic F van der Zwan J. Molecular architecture of the mouse nervous system. Cell. (2018) 174:999–1014 e22. doi: 10.1016/j.cell.2018.06.021 30096314 PMC6086934

[14] Drokhlyansky E Smillie CS Van Wittenberghe N Ericsson M Griffin GK Eraslan G. The human and mouse enteric nervous system at single-cell resolution. Cell. (2020) 182:1606–22 e23. doi: 10.1016/j.cell.2020.08.003 32888429 PMC8358727

[15] Guyer RA Stavely R Robertson K Bhave S Mueller JL Picard NM. Single-cell multiome sequencing clarifies enteric glial diversity and identifies an intraganglionic population poised for neurogenesis. Cell Rep. (2023) 42:112194. doi: 10.1016/j.celrep.2023.112194 36857184 PMC10123761

[16] Majd H Cesiulis A Samuel RM Richter MN Elder N Guyer RA. A call for a unified and multimodal definition of cellular identity in the enteric nervous system. bioRxiv. (2024). doi: 10.1101/2024.01.15.575794 PMC12528430 40954253

[17] Seguella L McClain JL Esposito G Gulbransen BD. Functional intraregional and interregional heterogeneity between myenteric glial cells of the colon and duodenum in mice. J Neurosci. (2022) 42:8694–708. doi: 10.1523/JNEUROSCI.2379-20.2022 PMC9671584 36319118

[18] Gershon MD Rothman TP. Enteric glia. Glia. (1991) 4:195–204. doi: 10.1002/glia.440040211 1827778

[19] Cabarrocas J Savidge TC Liblau RS. Role of enteric glial cells in inflammatory bowel disease. Glia. (2003) 41:81–93. doi: 10.1002/glia.10169 12465048

[20] Gulbransen BD. Enteric glia. San Rafael, California (USA): Biota Publishing (2014). doi: 10.4199/C00113ED1V01Y201407NGL002

[21] Ochoa-Cortes F Turco F Linan-Rico A Soghomonyan S Whitaker E Wehner S. Enteric glial cells: A new frontier in neurogastroenterology and clinical target for inflammatory bowel diseases. Inflammation Bowel Dis. (2016) 22:433–49. doi: 10.1097/MIB.0000000000000667 PMC4718179 26689598

[22] Boesmans W Rocha NP Reis HJ Holt M Vanden Berghe P. The astrocyte marker Aldh1L1 does not reliably label enteric glial cells. Neurosci Lett. (2014) 566:102–5. doi: 10.1016/j.neulet.2014.02.042 24589880

[23] Rao M Nelms BD Dong L Salinas-Rios V Rutlin M Gershon MD. Enteric glia express proteolipid protein 1 and are a transcriptionally unique population of glia in the mammalian nervous system. Glia. (2015) 63:2040–57. doi: 10.1002/glia.22876 PMC4695324 26119414

[24] Joseph NM He S Quintana E Kim YG Nunez G Morrison SJ. Enteric glia are multipotent in culture but primarily form glia in the adult rodent gut. J Clin Invest. (2011) 121:3398–411. doi: 10.1172/JCI58186 PMC3163971 21865643

[25] Laranjeira C Sandgren K Kessaris N Richardson W Potocnik A Vanden Berghe P. Glial cells in the mouse enteric nervous system can undergo neurogenesis in response to injury. J Clin Invest. (2011) 121:3412–24. doi: 10.1172/JCI58200 PMC3163972 21865647

[26] Ziegler AL Caldwell ML Craig SE Hellstrom EA Sheridan AE Touvron MS. Enteric glial cell network function is required for epithelial barrier restitution following intestinal ischemic injury in the early postnatal period. Am J Physiol Gastrointest Liver Physiol. (2024) 326:G228–G46. doi: 10.1152/ajpgi.00216.2022 PMC11211042 38147796

[27] Bauman BD Meng J Zhang L Louiselle A Zheng E Banerjee S. Enteric glial-mediated enhancement of intestinal barrier integrity is compromised by morphine. J Surg Res. (2017) 219:214–21. doi: 10.1016/j.jss.2017.05.099 PMC5708166 29078884

[28] Cheadle GA Costantini TW Bansal V Eliceiri BP Coimbra R. Cholinergic signaling in the gut: a novel mechanism of barrier protection through activation of enteric glia cells. Surg Infect (Larchmt). (2014) 15:387–93. doi: 10.1089/sur.2013.103 24828283

[29] Delvalle NM Dharshika C Morales-Soto W Fried DE Gaudette L Gulbransen BD. Communication between enteric neurons, glia, and nociceptors underlies the effects of tachykinins on neuroinflammation. Cell Mol Gastroenterol Hepatol. (2018) 6:321–44. doi: 10.1016/j.jcmgh.2018.05.009 PMC6091443 30116771

[30] Sundaresan S Meininger CA Kang AJ Photenhauer AL Hayes MM Sahoo N. Gastrin induces nuclear export and proteasome degradation of menin in enteric glial cells. Gastroenterology. (2017) 153:1555–67 e15. doi: 10.1053/j.gastro.2017.08.038 28859856 PMC5705278

[31] Vales S Bacola G Biraud M Touvron M Bessard A Geraldo F. Tumor cells hijack enteric glia to activate colon cancer stem cells and stimulate tumorigenesis. EBioMedicine. (2019) 49:172–88. doi: 10.1016/j.ebiom.2019.09.045 PMC6945247 31662289

[32] Selgrad M De Giorgio R Fini L Cogliandro RF Williams S Stanghellini V. JC virus infects the enteric glia of patients with chronic idiopathic intestinal pseudo-obstruction. Gut. (2009) 58:25–32. doi: 10.1136/gut.2008.152512 18593810 PMC2865195

[33] Rosenberg HJ Rao M. Enteric glia in homeostasis and disease: From fundamental biology to human pathology. iScience. (2021) 24:102863. doi: 10.1016/j.isci.2021.102863 34401661 PMC8348155

[34] Liu C Yang J. Enteric glial cells in immunological disorders of the gut. Front Cell Neurosci. (2022) 16:895871. doi: 10.3389/fncel.2022.895871 35573829 PMC9095930

[35] Scavuzzo MA Letai KC Maeno-Hikichi Y Wulftange WJ Shah IK Rameshbabu JS. Enteric glial hub cells coordinate intestinal motility. bioRxiv. (2023). doi: 10.1101/2023.06.07.544052

[36] Jessen KR Thorpe R Mirsky R. Molecular identity, distribution and heterogeneity of glial fibrillary acidic protein: an immunoblotting and immunohistochemical study of Schwann cells, satellite cells, enteric glia and astrocytes. J Neurocytol. (1984) 13:187–200. doi: 10.1007/BF01148114 6726286

[37] Park YM Chun H Shin JI Lee CJ. Astrocyte specificity and coverage of hGFAP-creERT2 [Tg(GFAP-cre/ERT2)13Kdmc] mouse line in various brain regions. Exp Neurobiol. (2018) 27:508–25. doi: 10.5607/en.2018.27.6.508 PMC6318562 30636902

[38] Zhang Z Ma Z Zou W Guo H Liu M Ma Y. The appropriate marker for astrocytes: comparing the distribution and expression of three astrocytic markers in different mouse cerebral regions. BioMed Res Int. (2019) 2019:9605265. doi: 10.1155/2019/9605265 31341912 PMC6613026

[39] Jessen KR Mirsky R. Glial fibrillary acidic polypeptides in peripheral glia. Molecular weight, heterogeneity and distribution. J Neuroimmunol. (1985) 8:377–93. doi: 10.1016/S0165-5728(85)80074-6 3891784

[40] Chanpong A Borrelli O Thapar N. Recent advances in understanding the roles of the enteric nervous system. Fac Rev. (2022) 11:7. doi: 10.12703/r 35373214 PMC8953438

[41] Grubisic V Gulbransen BD. Enteric glia: the most alimentary of all glia. J Physiol. (2017) 595:557–70. doi: 10.1113/JP271021 PMC5233670 27106597

[42] Delvalle NM Fried DE Rivera-Lopez G Gaudette L Gulbransen BD. Cholinergic activation of enteric glia is a physiological mechanism that contributes to the regulation of gastrointestinal motility. Am J Physiol Gastrointest Liver Physiol. (2018) 315:G473–G83. doi: 10.1152/ajpgi.00155.2018 PMC6230698 29927320

[43] Boesmans W Hao MM Fung C Li Z Van den Haute C Tack J. Structurally defined signaling in neuro-glia units in the enteric nervous system. Glia. (2019) 67:1167–78. doi: 10.1002/glia.23596 PMC6593736 30730592

[44] Le Berre C Naveilhan P Rolli-Derkinderen M. Enteric glia at center stage of inflammatory bowel disease. Neurosci Lett. (2023) 809:137315. doi: 10.1016/j.neulet.2023.137315 37257681

[45] Gabella G. Fine structure of the myenteric plexus in the Guinea-pig ileum. J Anat. (1972) 111:69–97. 4335909 PMC1271115

[46] Nasser Y Keenan CM Ma AC McCafferty DM Sharkey KA. Expression of a functional metabotropic glutamate receptor 5 on enteric glia is altered in states of inflammation. Glia. (2007) 55:859–72. doi: 10.1002/glia.20507 17405149

[47] Van Nassauw L Costagliola A Van Op den Bosch J Cecio A Vanderwinden JM Burnstock G. Region-specific distribution of the P2Y4 receptor in enteric glial cells and interstitial cells of Cajal within the Guinea-pig gastrointestinal tract. Auton Neurosci. (2006) 126–127:299–306. doi: 10.1016/j.autneu.2006.02.018 16616701

[48] Vanderwinden JM Timmermans JP Schiffmann SN. Glial cells, but not interstitial cells, express P2X7, an ionotropic purinergic receptor, in rat gastrointestinal musculature. Cell Tissue Res. (2003) 312:149–54. doi: 10.1007/s00441-003-0716-2 12684872

[49] Kimball BC Mulholland MW. Enteric glia exhibit P2U receptors that increase cytosolic calcium by a phospholipase C-dependent mechanism. J Neurochem. (1996) 66:604–12. doi: 10.1046/j.1471-4159.1996.66020604.x 8592130

[50] Zhang W Segura BJ Lin TR Hu Y Mulholland MW. Intercellular calcium waves in cultured enteric glia from neonatal Guinea pig. Glia. (2003) 42:252–62. doi: 10.1002/glia.10215 12673831

[51] Gulbransen BD Sharkey KA. Purinergic neuron-to-glia signaling in the enteric nervous system. Gastroenterology. (2009) 136:1349–58. doi: 10.1053/j.gastro.2008.12.058 19250649

[52] Gomes P Chevalier J Boesmans W Roosen L van den Abbeel V Neunlist M. ATP-dependent paracrine communication between enteric neurons and glia in a primary cell culture derived from embryonic mice. Neurogastroenterol Motil. (2009) 21:870–e62. doi: 10.1111/j.1365-2982.2009.01302.x 19368656

[53] Boesmans W Cirillo C Van den Abbeel V Van den Haute C Depoortere I Tack J. Neurotransmitters involved in fast excitatory neurotransmission directly activate enteric glial cells. Neurogastroenterol Motil. (2013) 25:e151–60. doi: 10.1111/nmo.12065 23279281

[54] Fung C Boesmans W Cirillo C Foong JPP Bornstein JC Vanden Berghe P. VPAC receptor subtypes tune purinergic neuron-to-glia communication in the murine submucosal plexus. Front Cell Neurosci. (2017) 11:118. doi: 10.3389/fncel.2017.00118 28487635 PMC5403822

[55] Gulbransen BD Bashashati M Hirota SA Gui X Roberts JA MacDonald JA. Activation of neuronal P2X7 receptor-pannexin-1 mediates death of enteric neurons during colitis. Nat Med. (2012) 18:600–4. doi: 10.1038/nm.2679 PMC3321107 22426419

[56] Fried DE Watson RE Robson SC Gulbransen BD. Ammonia modifies enteric neuromuscular transmission through glial gamma-aminobutyric acid signaling. Am J Physiol Gastrointest Liver Physiol. (2017) 313:G570–G80. doi: 10.1152/ajpgi.00154.2017 PMC5814673 28838986

[57] McClain J Grubisic V Fried D Gomez-Suarez RA Leinninger GM Sevigny J. Ca2+ responses in enteric glia are mediated by connexin-43 hemichannels and modulate colonic transit in mice. Gastroenterology. (2014) 146:497–507 e1. doi: 10.1053/j.gastro.2013.10.061 24211490 PMC3935238

[58] Murakami M Ohta T Otsuguro K Ito S. Involvement of prostaglandin E(2) derived from enteric glial cells in the action of bradykinin in cultured rat myenteric neurons. Neuroscience. (2007) 145:642–53. doi: 10.1016/j.neuroscience.2006.12.052 17275193

[59] Thomasi B Gulbransen B. Mini-review: Intercellular communication between enteric glia and neurons. Neurosci Lett. (2023) 806:137263. doi: 10.1016/j.neulet.2023.137263 37085112 PMC10150911

[60] Rao M Rastelli D Dong L Chiu S Setlik W Gershon MD. Enteric glia regulate gastrointestinal motility but are not required for maintenance of the epithelium in mice. Gastroenterology. (2017) 153:1068–81 e7. doi: 10.1053/j.gastro.2017.07.002 28711628 PMC5623141

[61] Grubisic V Gulbransen BD. Enteric glial activity regulates secretomotor function in the mouse colon but does not acutely affect gut permeability. J Physiol. (2017) 595:3409–24. doi: 10.1113/JP273492 PMC5451715 28066889

[62] Broadhead MJ Bayguinov PO Okamoto T Heredia DJ Smith TK. Ca2+ transients in myenteric glial cells during the colonic migrating motor complex in the isolated murine large intestine. J Physiol. (2012) 590:335–50. doi: 10.1113/jphysiol.2011.219519 PMC3285069 22063626

[63] Bush TG Savidge TC Freeman TC Cox HJ Campbell EA Mucke L. Fulminant jejuno-ileitis following ablation of enteric glia in adult transgenic mice. Cell. (1998) 93:189–201. doi: 10.1016/S0092-8674(00)81571-8 9568712

[64] Cornet A Savidge TC Cabarrocas J Deng WL Colombel JF Lassmann H. Enterocolitis induced by autoimmune targeting of enteric glial cells: a possible mechanism in Crohn's disease? Proc Natl Acad Sci U.S.A. (2001) 98:13306–11. doi: 10.1073/pnas.231474098 PMC60866 11687633

[65] Baghdadi MB Ayyaz A Coquenlorge S Chu B Kumar S Streutker C. Enteric glial cell heterogeneity regulates intestinal stem cell niches. Cell Stem Cell. (2022) 29:86–100 e6. doi: 10.1016/j.stem.2021.10.004 34727519

[66] Progatzky F Pachnis V. Enteric glia bring fresh WNT to the intestinal stem cell niche. Cell Stem Cell. (2022) 29:3–4. doi: 10.1016/j.stem.2021.12.003 34995494

[67] Meir M Flemming S Burkard N Wagner J Germer CT Schlegel N. The glial cell-line derived neurotrophic factor: a novel regulator of intestinal barrier function in health and disease. Am J Physiol Gastrointest Liver Physiol. (2016) 310:G1118–23. doi: 10.1152/ajpgi.00125.2016 27151942

[68] Bohorquez DV Samsa LA Roholt A Medicetty S Chandra R Liddle RA. An enteroendocrine cell-enteric glia connection revealed by 3D electron microscopy. PloS One. (2014) 9:e89881. doi: 10.1371/journal.pone.0089881 24587096 PMC3935946

[69] Neunlist M Aubert P Bonnaud S Van Landeghem L Coron E Wedel T. Enteric glia inhibit intestinal epithelial cell proliferation partly through a TGF-beta1-dependent pathway. Am J Physiol Gastrointest Liver Physiol. (2007) 292:G231–41. doi: 10.1152/ajpgi.00276.2005 16423922

[70] Van Landeghem L Chevalier J Mahe MM Wedel T Urvil P Derkinderen P. Enteric glia promote intestinal mucosal healing via activation of focal adhesion kinase and release of proEGF. Am J Physiol Gastrointest Liver Physiol. (2011) 300:G976–87. doi: 10.1152/ajpgi.00427.2010 PMC3119120 21350188

[71] Dora D Ferenczi S Stavely R Toth VE Varga ZV Kovacs T. Evidence of a myenteric plexus barrier and its macrophage-dependent degradation during murine colitis: implications in enteric neuroinflammation. Cell Mol Gastroenterol Hepatol. (2021) 12:1617–41. doi: 10.1016/j.jcmgh.2021.07.003 PMC8551790 34246810

[72] Kabouridis PS Lasrado R McCallum S Chng SH Snippert HJ Clevers H. Microbiota controls the homeostasis of glial cells in the gut lamina propria. Neuron. (2015) 85:289–95. doi: 10.1016/j.neuron.2014.12.037 PMC4306542 25578362

[73] Vicentini FA Keenan CM Wallace LE Woods C Cavin JB Flockton AR. Intestinal microbiota shapes gut physiology and regulates enteric neurons and glia. Microbiome. (2021) 9:210. doi: 10.1186/s40168-021-01165-z 34702353 PMC8549243

[74] Inlender T Nissim-Eliraz E Stavely R Hotta R Goldstein AM Yagel S. Homeostasis of mucosal glial cells in human gut is independent of microbiota. Sci Rep. (2021) 11:12796. doi: 10.1038/s41598-021-92384-9 34140608 PMC8211706

[75] De Schepper S Verheijden S Aguilera-Lizarraga J Viola MF Boesmans W Stakenborg N. Self-maintaining gut macrophages are essential for intestinal homeostasis. Cell. (2018) 175:400–15 e13. doi: 10.1016/j.cell.2018.07.048 30173915

[76] Obata Y Castano A Boeing S Bon-Frauches AC Fung C Fallesen T. Neuronal programming by microbiota regulates intestinal physiology. Nature. (2020) 578:284–9. doi: 10.1038/s41586-020-1975-8 32025031

[77] Drossman DA. Functional gastrointestinal disorders: history, pathophysiology, clinical features and rome IV. Gastroenterology. (2016) 150(6):1262–79.e2. doi: 10.1053/j.gastro.2016.02.032 27144617

[78] Phillips RJ Kieffer EJ Powley TL. Loss of glia and neurons in the myenteric plexus of the aged Fischer 344 rat. Anat Embryol (Berl). (2004) 209:19–30. doi: 10.1007/s00429-004-0426-x 15480773

[79] Camilleri M Cowen T Koch TR. Enteric neurodegeneration in ageing. Neurogastroenterol Motil. (2008) 20:418–29. doi: 10.1111/j.1365-2982.2008.01134.x 18371012

[80] Saffrey MJ. Aging of the mammalian gastrointestinal tract: a complex organ system. Age (Dordr). (2014) 36:9603. doi: 10.1007/s11357-013-9603-2 24352567 PMC4082571

[81] Bon-Frauches AC Boesmans W. The enteric nervous system: the hub in a star network. Nat Rev Gastroenterol Hepatol. (2020) 17:717–8. doi: 10.1038/s41575-020-00377-2 33087897

[82] Rosenbaum C Schick MA Wollborn J Heider A Scholz CJ Cecil A. Activation of myenteric glia during acute inflammation *in vitro* and in vivo. PloS One. (2016) 11:e0151335. doi: 10.1371/journal.pone.0151335 26964064 PMC4786261

[83] Progatzky F Pachnis V. The role of enteric glia in intestinal immunity. Curr Opin Immunol. (2022) 77:102183. doi: 10.1016/j.coi.2022.102183 35533467 PMC9586875

[84] Pochard C Coquenlorge S Freyssinet M Naveilhan P Bourreille A Neunlist M. The multiple faces of inflammatory enteric glial cells: is Crohn's disease a gliopathy? Am J Physiol Gastrointest Liver Physiol. (2018) 315:G1–G11. doi: 10.1152/ajpgi.00016.2018 29517926

[85] Scott NA Beart RW Jr. Weiland LH Cha SS Lieber MM. Carcinoma of the anal canal and flow cytometric DNA analysis. Br J Cancer. (1989) 60:56–8. doi: 10.1038/bjc.1989.219 PMC2247343 2803916

[86] Linan-Rico A Turco F Ochoa-Cortes F Harzman A Needleman BJ Arsenescu R. Molecular signaling and dysfunction of the human reactive enteric glial cell phenotype: implications for GI infection, IBD, POI, neurological, motility, and GI disorders. Inflammation Bowel Dis. (2016) 22:1812–34. doi: 10.1097/MIB.0000000000000854 PMC4993196 27416040

[87] Progatzky F Shapiro M Chng SH Garcia-Cassani B Classon CH Sevgi S. Regulation of intestinal immunity and tissue repair by enteric glia. Nature. (2021) 599:125–30. doi: 10.1038/s41586-021-04006-z PMC7612231 34671159

[88] Escartin C Galea E Lakatos A O'Callaghan JP Petzold GC Serrano-Pozo A. Reactive astrocyte nomenclature, definitions, and future directions. Nat Neurosci. (2021) 24:312–25. doi: 10.1038/s41593-020-00783-4 PMC8007081 33589835

[89] Ibiza S Garcia-Cassani B Ribeiro H Carvalho T Almeida L Marques R. Glial-cell-derived neuroregulators control type 3 innate lymphoid cells and gut defence. Nature. (2016) 535:440–3. doi: 10.1038/nature18644 PMC4962913 27409807

[90] Grubisic V McClain JL Fried DE Grants I Rajasekhar P Csizmadia E. Enteric glia modulate macrophage phenotype and visceral sensitivity following inflammation. Cell Rep. (2020) 32:108100. doi: 10.1016/j.celrep.2020.108100 32905782 PMC7518300

[91] Chow AK Grubisic V Gulbransen BD. Enteric glia regulate lymphocyte activation *via* autophagy-mediated MHC-II expression. Cell Mol Gastroenterol Hepatol. (2021) 12:1215–37. doi: 10.1016/j.jcmgh.2021.06.008 PMC8449089 34166814

[92] Fettucciari K Ponsini P Gioe D Macchioni L Palumbo C Antonelli E. Enteric glial cells are susceptible to Clostridium difficile toxin B. Cell Mol Life Sci. (2017) 74:1527–51. doi: 10.1007/s00018-016-2426-4 PMC11107567 27891552

[93] Macchioni L Davidescu M Fettucciari K Petricciuolo M Gatticchi L Gioe D. Enteric glial cells counteract Clostridium difficile Toxin B through a NADPH oxidase/ROS/JNK/caspase-3 axis, without involving mitochondrial pathways. Sci Rep. (2017) 7:45569. doi: 10.1038/srep45569 28349972 PMC5368562

[94] Fettucciari K Marguerie F Fruganti A Marchegiani A Spaterna A Brancorsini S. Clostridioides difficile toxin B alone and with pro-inflammatory cytokines induces apoptosis in enteric glial cells by activating three different signalling pathways mediated by caspases, calpains and cathepsin B. Cell Mol Life Sci. (2022) 79:442. doi: 10.1007/s00018-022-04459-z 35864342 PMC9304068

[95] Loureiro AV Moura-Neto LI Martins CS Silva PIM Lopes MBS Leitao RFC. Role of Pannexin-1-P2X7R signaling on cell death and pro-inflammatory mediator expression induced by Clostridioides difficile toxins in enteric glia. Front Immunol. (2022) 13:956340. doi: 10.3389/fimmu.2022.956340 36072579 PMC9442043

[96] Costa DVS Shin JH Goldbeck SM Bolick DT Mesquita FS Loureiro AV. Adenosine receptors differentially mediate enteric glial cell death induced by Clostridioides difficile Toxins A and B. Front Immunol. (2022) 13:956326. doi: 10.3389/fimmu.2022.956326 36726986 PMC9885079

[97] Costa DVS Moura-Neto V Bolick DT Guerrant RL Fawad JA Shin JH. S100B inhibition attenuates intestinal damage and diarrhea severity during clostridioides difficile infection by modulating inflammatory response. Front Cell Infect Microbiol. (2021) 11:739874. doi: 10.3389/fcimb.2021.739874 34568098 PMC8461106

[98] Schneider LCL do Nascimento JCP Trevizan AR Gois MB Borges SC Beraldi EJ. Toxoplasma gondii promotes changes in VIPergic submucosal neurons, mucosal intraepithelial lymphocytes, and goblet cells during acute infection in the ileum of rats. Neurogastroenterol Motil. (2018) 30:e13264. doi: 10.1111/nmo.13264 29266818

[99] Trevizan AR Schneider LCL Araujo EJA Garcia JL Buttow NC Nogueira-Melo GA. Acute Toxoplasma gondii infection alters the number of neurons and the proportion of enteric glial cells in the duodenum in Wistar rats. Neurogastroenterol Motil. (2019) 31:e13523. doi: 10.1111/nmo.13523 30537037

[100] Dennehy PH. Rotavirus infection: A disease of the past? Infect Dis Clin North Am. (2015) 29:617–35. doi: 10.1016/j.idc.2015.07.002 26337738

[101] Serrander R Magnusson KE Sundqvist T. Acute infections with Giardia lamblia and rotavirus decrease intestinal permeability to low-molecular weight polyethylene glycols (PEG 400). Scand J Infect Dis. (1984) 16:339–44. doi: 10.3109/00365548409073958 6528222

[102] Johansen K Stintzing G Magnusson KE Sundqvist T Jalil F Murtaza A. Intestinal permeability assessed with polyethylene glycols in children with diarrhea due to rotavirus and common bacterial pathogens in a developing community. J Pediatr Gastroenterol Nutr. (1989) 9:307–13. doi: 10.1002/j.1536-4801.1989.tb09875.x 2693681

[103] Hagbom M De Faria FM Winberg ME Westerberg S Nordgren J Sharma S. Neurotrophic factors protect the intestinal barrier from rotavirus insult in mice. mBio. (2020) 11:e02834–19. doi: 10.1128/mBio.02834-19 PMC6974565 31964731

[104] Stintzing G Johansen K Magnusson KE Svensson L Sundqvist T. Intestinal permeability in small children during and after rotavirus diarrhoea assessed with different-size polyethyleneglycols (PEG 400 and PEG 1000). Acta Paediatr Scand. (1986) 75:1005–9. doi: 10.1111/j.1651-2227.1986.tb10331.x 3564957

[105] Istrate C Hagbom M Vikstrom E Magnusson KE Svensson L. Rotavirus infection increases intestinal motility but not permeability at the onset of diarrhea. J Virol. (2014) 88:3161–9. doi: 10.1128/JVI.02927-13 PMC3957942 24371070

[106] Giuffre M Moretti R Campisciano G da Silveira ABM Monda VM Comar M. You talking to me? Says the enteric nervous system (ENS) to the microbe. How intestinal microbes interact with the ENS. J Clin Med. (2020) 9:3705. doi: 10.3390/jcm9113705 33218203 PMC7699249

[107] Westerberg S Hagbom M Rajan A Loitto V Persson BD Allard A. Interaction of human enterochromaffin cells with human enteric adenovirus 41 leads to serotonin release and subsequent activation of enteric glia cells. J Virol. (2018) 92:e00026–18. doi: 10.1128/JVI.00026-18 PMC5972892 29367250

[108] Grundmann D Loris E Maas-Omlor S Huang W Scheller A Kirchhoff F. Enteric glia: S100, GFAP, and beyond. Anat Rec (Hoboken). (2019) 302:1333–44. doi: 10.1002/ar.24128 30951262

[109] Esposito G Capoccia E Gigli S Pesce M Bruzzese E D'Alessandro A. HIV-1 Tat-induced diarrhea evokes an enteric glia-dependent neuroinflammatory response in the central nervous system. Sci Rep. (2017) 7:7735. doi: 10.1038/s41598-017-05245-9 28798420 PMC5552820

[110] Jantsch J Chikkaballi D Hensel M. Cellular aspects of immunity to intracellular Salmonella enterica. Immunol Rev. (2011) 240:185–95. doi: 10.1111/j.1600-065X.2010.00981.x 21349094

[111] Wang H Foong JPP Harris NL Bornstein JC. Enteric neuroimmune interactions coordinate intestinal responses in health and disease. Mucosal Immunol. (2022) 15:27–39. doi: 10.1038/s41385-021-00443-1 34471248 PMC8732275

[112] Meira de-Faria F Casado-Bedmar M Marten Lindqvist C Jones MP Walter SA Keita AV. Altered interaction between enteric glial cells and mast cells in the colon of women with irritable bowel syndrome. Neurogastroenterol Motil. (2021) 33:e14130. doi: 10.1111/nmo.14130 33797165

[113] Ciccarelli S Stolfi I Caramia G. Management strategies in the treatment of neonatal and pediatric gastroenteritis. Infect Drug Resist. (2013) 6:133–61. doi: 10.2147/IDR PMC3815002 24194646

[114] Kartau M Verkkoniemi-Ahola A Paetau A Palomaki M Janes R Ristola M. The incidence and predisposing factors of john cunningham virus-induced progressive multifocal leukoencephalopathy in southern Finland: A population-based study. Open Forum Infect Dis. (2019) 6:ofz024. doi: 10.1093/ofid/ofz024 30815501 PMC6386113

[115] Beltrami S Gordon J. Immune surveillance and response to JC virus infection and PML. J Neurovirol. (2014) 20:137–49. doi: 10.1007/s13365-013-0222-6 PMC3972311 24297501

[116] Esposito G Pesce M Seguella L Sanseverino W Lu J Sarnelli G. Can the enteric nervous system be an alternative entrance door in SARS-CoV2 neuroinvasion? Brain Behav Immun. (2020) 87:93–4. doi: 10.1016/j.bbi.2020.04.060 PMC7179488 32335192

[117] Sambataro G Giuffre M Sambataro D Palermo A Vignigni G Cesareo R. The model for early COvid-19 recognition (MECOR) score: A proof-of-concept for a simple and low-cost tool to recognize a possible viral etiology in community-acquired pneumonia patients during COVID-19 outbreak. Diagnostics (Basel). (2020) 10:619. doi: 10.3390/diagnostics10090619 32825763 PMC7555441

[118] Giuffre M Di Bella S Sambataro G Zerbato V Cavallaro M Occhipinti AA. COVID-19-induced thrombosis in patients without gastrointestinal symptoms and elevated fecal calprotectin: hypothesis regarding mechanism of intestinal damage associated with COVID-19. Trop Med Infect Dis. (2020) 5:147. doi: 10.3390/tropicalmed5030147 32947803 PMC7557761

[119] Giuffre M Bozzato AM Di Bella S Occhipinti AA Martingano P Cavallaro MFM. Spontaneous rectal perforation in a patient with SARS-coV-2 infection. J Pers Med. (2020) 10:157. doi: 10.3390/jpm10040157 33049924 PMC7712943

[120] Stakenborg M Abdurahiman S De Simone V Goverse G Stakenborg N van Baarle L. Enteric glial cells favor accumulation of anti-inflammatory macrophages during the resolution of muscularis inflammation. Mucosal Immunol. (2022) 15:1296–308. doi: 10.1038/s41385-022-00563-2 PMC9705256 36071145

[121] Kermarrec L Durand T Neunlist M Naveilhan P Neveu I. Enteric glial cells have specific immunosuppressive properties. J Neuroimmunol. (2016) 295–296:79–83. doi: 10.1016/j.jneuroim.2016.04.011 27235353

[122] Schneider R Leven P Glowka T Kuzmanov I Lysson M Schneiker B. A novel P2X2-dependent purinergic mechanism of enteric gliosis in intestinal inflammation. EMBO Mol Med. (2021) 13:e12724. doi: 10.15252/emmm.202012724 33332729 PMC7799361

[123] Stoffels B Hupa KJ Snoek SA van Bree S Stein K Schwandt T. Postoperative ileus involves interleukin-1 receptor signaling in enteric glia. Gastroenterology. (2014) 146:176–87 e1. doi: 10.1053/j.gastro.2013.09.030 24067878

[124] Hupa KJ Stein K Schneider R Lysson M Schneiker B Hornung V. AIM2 inflammasome-derived IL-1beta induces postoperative ileus in mice. Sci Rep. (2019) 9:10602. doi: 10.1038/s41598-019-46968-1 31332247 PMC6646358

[125] Schneider R Leven P Mallesh S Bresser M Schneider L Mazzotta E. IL-1-dependent enteric gliosis guides intestinal inflammation and dysmotility and modulates macrophage function. Commun Biol. (2022) 5:811. doi: 10.1038/s42003-022-03772-4 35962064 PMC9374731

[126] Collaborators GBDIBD. The global, regional, and national burden of inflammatory bowel disease in 195 countries and territories, 1990–2017: a systematic analysis for the Global Burden of Disease Study 2017. Lancet Gastroenterol Hepatol. (2020) 5:17–30. doi: 10.1016/S2468-1253(19)30333-4 31648971 PMC7026709

[127] Baumgart DC Carding SR. Inflammatory bowel disease: cause and immunobiology. Lancet. (2007) 369:1627–40. doi: 10.1016/S0140-6736(07)60750-8 17499605

[128] Sgambato D Miranda A Ranaldo R Federico A Romano M. The role of stress in inflammatory bowel diseases. Curr Pharm Des. (2017) 23:3997–4002. doi: 10.2174/1381612823666170228123357 28245757

[129] Sun Y Li L Xie R Wang B Jiang K Cao H. Stress triggers flare of inflammatory bowel disease in children and adults. Front Pediatr. (2019) 7:432. doi: 10.3389/fped.2019.00432 31709203 PMC6821654

[130] Araki M Shinzaki S Yamada T Arimitsu S Komori M Shibukawa N. Psychologic stress and disease activity in patients with inflammatory bowel disease: A multicenter cross-sectional study. PloS One. (2020) 15:e0233365. doi: 10.1371/journal.pone.0233365 32453762 PMC7250441

[131] Schneider KM Blank N Alvarez Y Thum K Lundgren P Litichevskiy L. The enteric nervous system relays psychological stress to intestinal inflammation. Cell. (2023) 186:2823–38 e20. doi: 10.1016/j.cell.2023.05.001 37236193 PMC10330875

[132] von Boyen GB Schulte N Pfluger C Spaniol U Hartmann C Steinkamp M. Distribution of enteric glia and GDNF during gut inflammation. BMC Gastroenterol. (2011) 11:3. doi: 10.1186/1471-230X-11-3 21235736 PMC3034687

[133] Cirillo C Sarnelli G Esposito G Grosso M Petruzzelli R Izzo P. Increased mucosal nitric oxide production in ulcerative colitis is mediated in part by the enteroglial-derived S100B protein. Neurogastroenterol Motil. (2009) 21:1209–e112. doi: 10.1111/j.1365-2982.2009.01346.x 19558426

[134] Esposito G Capoccia E Turco F Palumbo I Lu J Steardo A. Palmitoylethanolamide improves colon inflammation through an enteric glia/toll like receptor 4-dependent PPAR-alpha activation. Gut. (2014) 63:1300–12. doi: 10.1136/gutjnl-2013-305005 24082036

[135] Villanacci V Bassotti G Nascimbeni R Antonelli E Cadei M Fisogni S. Enteric nervous system abnormalities in inflammatory bowel diseases. Neurogastroenterol Motil. (2008) 20:1009–16. doi: 10.1111/j.1365-2982.2008.01146.x 18492026

[136] Cirillo C Sarnelli G Esposito G Turco F Steardo L Cuomo R. S100B protein in the gut: the evidence for enteroglial-sustained intestinal inflammation. World J Gastroenterol. (2011) 17:1261–6. doi: 10.3748/wjg.v17.i10.1261 PMC3068260 21455324

[137] Muller PA Koscso B Rajani GM Stevanovic K Berres ML Hashimoto D. Crosstalk between muscularis macrophages and enteric neurons regulates gastrointestinal motility. Cell. (2014) 158:300–13. doi: 10.1016/j.cell.2014.04.050 PMC4149228 25036630

[138] Spits H Artis D Colonna M Diefenbach A Di Santo JP Eberl G. Innate lymphoid cells–a proposal for uniform nomenclature. Nat Rev Immunol. (2013) 13:145–9. doi: 10.1038/nri3365 23348417

[139] Spits H Di Santo JP. The expanding family of innate lymphoid cells: regulators and effectors of immunity and tissue remodeling. Nat Immunol. (2011) 12:21–7. doi: 10.1038/ni.1962 21113163

[140] Eberl G. Development and evolution of RORgammat+ cells in a microbe's world. Immunol Rev. (2012) 245:177–88. doi: 10.1111/j.1600-065X.2011.01071.x 22168420

[141] Artis D Spits H. The biology of innate lymphoid cells. Nature. (2015) 517:293–301. doi: 10.1038/nature14189 25592534

[142] Savidge TC Newman P Pothoulakis C Ruhl A Neunlist M Bourreille A. Enteric glia regulate intestinal barrier function and inflammation *via* release of S-nitrosoglutathione. Gastroenterology. (2007) 132:1344–58. doi: 10.1053/j.gastro.2007.01.051 17408650

[143] Chen CB Tahboub F Plesec T Kay M Radhakrishnan K. A review of autoimmune enteropathy and its associated syndromes. Dig Dis Sci. (2020) 65:3079–90. doi: 10.1007/s10620-020-06540-8 32833153

[144] Li Z Zhang X Zhou H Liu W Li J. Exogenous S-nitrosoglutathione attenuates inflammatory response and intestinal epithelial barrier injury in endotoxemic rats. J Trauma Acute Care Surg. (2016) 80:977–84. doi: 10.1097/TA.0000000000001008 26891162

[145] Xie Q Chen X Meng ZM Huang XL Zhang Q Zhou JQ. Glial-derived neurotrophic factor regulates enteric mast cells and ameliorates dextran sulfate sodium-induced experimental colitis. Int Immunopharmacol. (2020) 85:106638. doi: 10.1016/j.intimp.2020.106638 32470881

[146] Li H Fan C Lu H Feng C He P Yang X. Protective role of berberine on ulcerative colitis through modulating enteric glial cells-intestinal epithelial cells-immune cells interactions. Acta Pharm Sin B. (2020) 10:447–61. doi: 10.1016/j.apsb.2019.08.006 PMC7049614 32140391

[147] Vaes N Idris M Boesmans W Alves MM Melotte V. Nerves in gastrointestinal cancer: from mechanism to modulations. Nat Rev Gastroenterol Hepatol. (2022) 19:768–84. doi: 10.1038/s41575-022-00669-9 36056202

[148] Liebig C Ayala G Wilks JA Berger DH Albo D. Perineural invasion in cancer: a review of the literature. Cancer. (2009) 115:3379–91. doi: 10.1002/cncr.24396 19484787

[149] Knijn N Mogk SC Teerenstra S Simmer F Nagtegaal ID. Perineural invasion is a strong prognostic factor in colorectal cancer: A systematic review. Am J Surg Pathol. (2016) 40:103–12. doi: 10.1097/PAS.0000000000000518 26426380

[150] Liebig C Ayala G Wilks J Verstovsek G Liu H Agarwal N. Perineural invasion is an independent predictor of outcome in colorectal cancer. J Clin Oncol. (2009) 27:5131–7. doi: 10.1200/JCO.2009.22.4949 PMC2773472 19738119

[151] Purandare NC Dua SG Arora A Shah S Rangarajan V. Colorectal cancer - patterns of locoregional recurrence and distant metastases as demonstrated by FDG PET / CT. Indian J Radiol Imaging. (2010) 20:284–8. doi: 10.4103/0971-3026.73545 PMC3056626 21423904

[152] Mariant CL Bacola G Van Landeghem L. Mini-Review: Enteric glia of the tumor microenvironment: An affair of corruption. Neurosci Lett. (2023) 814:137416. doi: 10.1016/j.neulet.2023.137416 37572875 PMC10967235

[153] Godlewski J Kmiec Z. Colorectal cancer invasion and atrophy of the enteric nervous system: potential feedback and impact on cancer progression. Int J Mol Sci. (2020) 21:3391. doi: 10.3390/ijms21093391 32403316 PMC7247003

[154] Ciurea RN Rogoveanu I Pirici D Tartea GC Streba CT Florescu C. B2 adrenergic receptors and morphological changes of the enteric nervous system in colorectal adenocarcinoma. World J Gastroenterol. (2017) 23:1250–61. doi: 10.3748/wjg.v23.i7.1250 PMC5323450 28275305

[155] Duchalais E Guilluy C Nedellec S Touvron M Bessard A Touchefeu Y. Colorectal cancer cells adhere to and migrate along the neurons of the enteric nervous system. Cell Mol Gastroenterol Hepatol. (2018) 5:31–49. doi: 10.1016/j.jcmgh.2017.10.002 29188232 PMC5696385

[156] Vaes N Schonkeren SL Rademakers G Holland AM Koch A Gijbels MJ. Loss of enteric neuronal Ndrg4 promotes colorectal cancer *via* increased release of Nid1 and Fbln2. EMBO Rep. (2021) 22:e51913. doi: 10.15252/embr.202051913 33890711 PMC8183412

[157] Neunlist M Van Landeghem L Bourreille A Savidge T. Neuro-glial crosstalk in inflammatory bowel disease. J Intern Med. (2008) 263:577–83. doi: 10.1111/j.1365-2796.2008.01963.x 18479256

[158] Yuan R Bhattacharya N Kenkel JA Shen J DiMaio MA Bagchi S. Enteric glia play a critical role in promoting the development of colorectal cancer. Front Oncol. (2020) 10:595892. doi: 10.3389/fonc.2020.595892 33282743 PMC7691584

[159] Seguella L Rinaldi F Marianecci C Capuano R Pesce M Annunziata G. Pentamidine niosomes thwart S100B effects in human colon carcinoma biopsies favouring wtp53 rescue. J Cell Mol Med. (2020) 24:3053–63. doi: 10.1111/jcmm.14943 PMC7077541 32022398

[160] Baarle Lv Simone VD Schneider L Santhosh S Abdurahiman S Biscu F. IL-1R signaling drives enteric glia-macrophage interactions in colorectal cancer. bioRxiv. (2023). doi: 10.1101/2023.06.01.543246 PMC11271635 39030280

[161] Duan S Sawyer TW Sontz RA Wieland BA Diaz AF Merchant JL. GFAP-directed inactivation of men1 exploits glial cell plasticity in favor of neuroendocrine reprogramming. Cell Mol Gastroenterol Hepatol. (2022) 14:1025–51. doi: 10.1016/j.jcmgh.2022.06.009 PMC9490044 35835391

